# *In vivo* effects of single or combined topical neuroprotective and regenerative agents on degeneration of retinal ganglion cells in rat optic nerve crush model

**DOI:** 10.1038/s41598-018-36473-2

**Published:** 2019-01-14

**Authors:** Yuta Kitamura, Guzel Bikbova, Takayuki Baba, Shuichi Yamamoto, Toshiyuki Oshitari

**Affiliations:** 1 Department of Ophthalmology and Visual Science Chiba University, Graduate School of Medicine, Inohana 1-8-1, Chuo-ku, Chiba, 260-8670 Chiba Japan; 2 Department of Ophthalmology, International University of Health and Welfare, School of Medicine, Kouzunomori 4-3, Narita, 286-8686 Chiba Japan

## Abstract

To determine the effectiveness of a single or a combination of topical neurotrophic factors (NFs) in protecting retinal ganglion cells (RGCs) in the rat optic nerve crush (ONC) model, the left ONC was performed to induce the death of the RGCs in adult Sprague-Dawley rats. The NFs studied were tauroursodeoxycholic acid (TUDCA), citicoline, neurotrophin-4 (NT-4), combined TUDCA/citicoline (Doublet-1), combined TUDCA/NT-4 (Doublet-2), combined TUDCA/citicoline/NT-4 (Triplet), and PBS. After 2 weeks, the number of RGCs was determined by Brn3a immunostaining. The optic nerves were immunostained for anti-Growth Associated Protein-43(GAP-43) and -200kD neurofilament heavy antibody to study optic nerve regeneration. Two weeks after the ONC, the densities of RGCs in all treated eyes were significantly higher than that of the PBS treated eyes. In the Triplet group, the number of RGC axons after ONC was significantly higher than that in all of the single treatment groups and the number of TUNEL positive cells was significantly reduced and the number of GAP-43 immunopositive axons was significantly greater than those in the PBS group. Neovascularization was observed only in the Doublet-1 group. We conclude that the combination of the three NFs was the most effective way to protect RGCs after the ONC.

## Introduction

There is an accumulation of glycated proteins, lipids, and nucleic acid with increasing age which results from the elevated blood glucose. These changes increase the oxidative stress which then triggers further modifications of the proteins, and the development of neuronal and vascular complications^1^. The results of our recent study showed that glycation plays a role in the pathogenesis of retinal diabetic neuropathy, and it acts by triggering different mechanisms resulting in neuronal dysfunction^2–6^. Caspase-dependent and caspase-independent cell death pathways were confirmed to be involved in the apoptosis of retinal ganglion cells (RGCs) in the presence of very low doses of advanced glycation end products (AGEs)^4^. Additionally, an expression of the receptors of the AGE (*RAGE*) genes indicated that there was an increase in the expression of transcription factors NF-kB and SP1 in AGEs-exposed retinas^5^. These findings suggested that a strategy for treating neurodegenerative diseases might be obtained by determining the genes and pathways involved in neurodegeneration.

We determined earlier that neuroprotective and regenerative factors such as neurotrophin-4 (NT-4)^4,7^, citicoline^8^, tauroursodeoxycholic acid (TUDCA)^5^, and their combinations enhanced the regeneration of neurites in AGEs-exposed retinas *in vitro*^9^. These findings suggested that they were axon-protective agents that could be used to treat neuronal diseases associated with AGEs accumulation. The results showed that combinations of citicoline and TUDCA (doublet) and citicoline, TUDCA, and NT-4 (triplet) significantly increased number of regenerated neurites and decreased the number of TUNEL-positive cells. These changes were correlated with a decrease in the expressions of caspase-9 and JNK^9^ indicating the neuroprotective abilities of these agents on cultured retinal cells exposed to AGEs^9^.

The delivery of neurotrophic factors to the RGCs safely, effectively, and for long durations would be best accomplished by topical applications. Several studies have been performed using a single neurotrophic factor applied topically to test its neuroprotective effect^10,11^. The results indicated that citicoline eyedrops can enhance the visual function of patients with glaucoma without adverse side effects^12,13^. Another recent study evaluated the neuroprotective effect of topical citicoline in a mouse model of diabetic retinopathy^14^. The results indicated that topical citicoline prevented the reduction of the retinal thickness in diabetic retinas^14^. However, very few attempts have been made using combinations of different neurotrophic factors in *in vivo* studies.

Thus, the purpose of this study was to determine the neuroprotective and regenerative effects of topical administrations of three neurotrophic factors, viz., citicoline, TUDCA, NT-4, used individually or in combinations. To accomplish this, the rat optic nerve crush (ONC) model was used because it is an easy technique to use for a short period and our prior experience with this model^15,16^. Furthermore, the mechanisms of the RGC death after optic nerve injuries^17–20^ are, in part, common with those in our culture system or in degenerating neurons^2,4–6,9^ of human diabetic retinas^3,21^.

## Results

### Brn3a immunopositivity in retinal flat mounts

**Figure 1:**
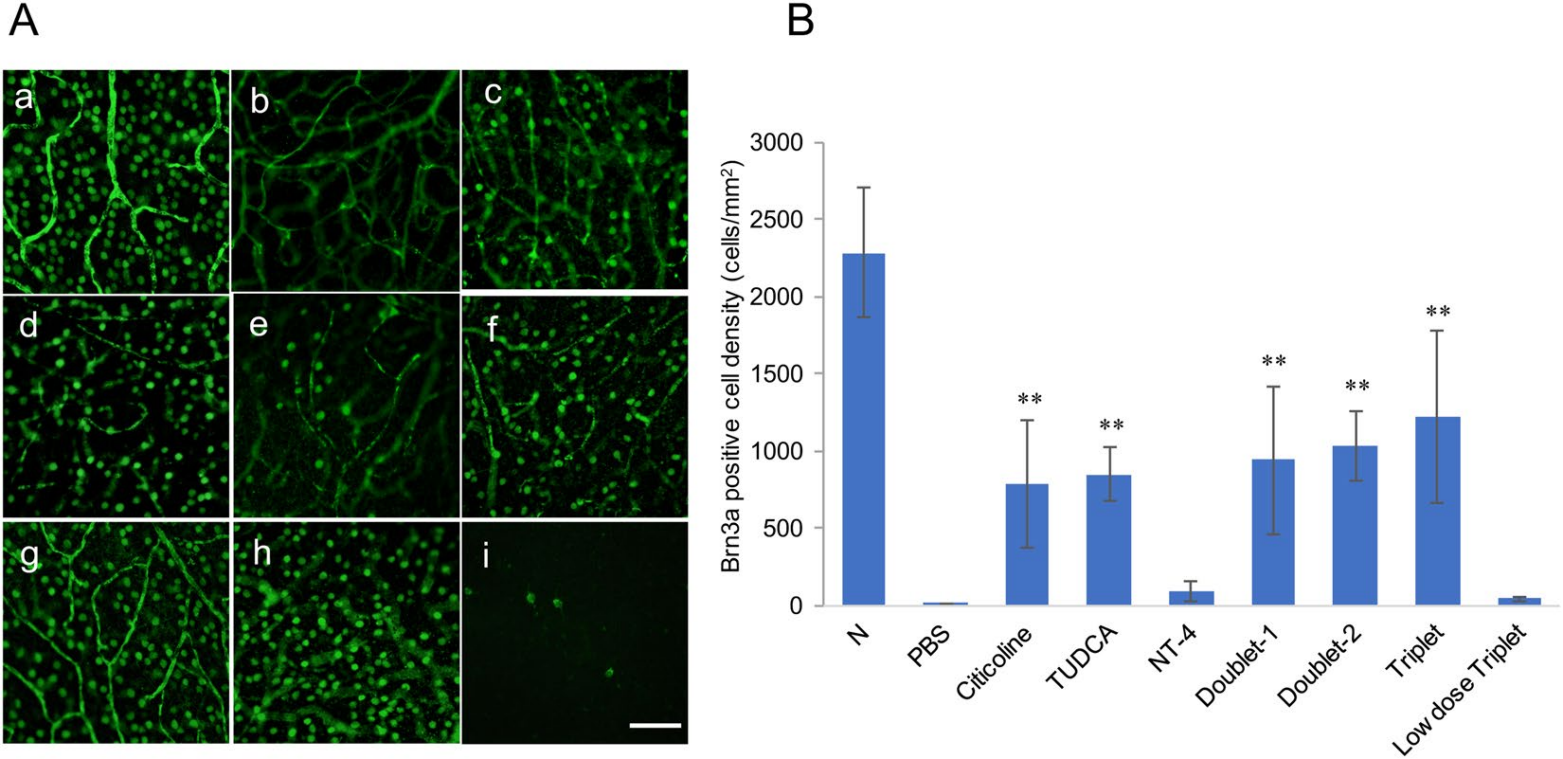
(**A**) Representative photomicrographs of Brn3a immunopositive cells in the flat mount retina. (a) Normal, (b) PBS (ONC + PBS), (c) citicoline (ONC + citicoline), (d) TUDCA (ONC + TUDCA), (e) NT-4 (ONC + NT-4), (f) Doublet-1 (ONC + citicoline + TUDCA), (g) Doublet-2 (ONC + TUDCA + NT-4), (h) Triplet (ONC + citicoline + TUDCA + NT4). (i) Low dose Triplet (Scale Bar: 50 μm.) (**B**) Graph showing RGC count (cells/mm^2^) of each group two weeks after ONC. The densities of RGCs in all of the eyes except NT-4 group were significantly higher than those of RGCs without treatment. (**P < 0.001) Values represent means ± SEM of n = 6 animals per experimental group. Significances of intergroup differences were evaluated by one-way ANOVA with Tukey’s multiple comparisons test.

To assess the neuroprotective effects of the topical neurotrophic factors, we determined the number of surviving RGCs two weeks after the optic nerve was crushed. This was done by immunostaining the RGCs with Brn3a, a member of the POU-domain transcription factors which is expressed in RGC. The density, cell number/mm^2^, of the RGCs was 4.75 ± 6.63/mm^2^ in the retina without any treatment, which was significantly fewer than the 2280 ± 416.1/mm^2^ in the normal control (*P* < 0.001). In addition to non-operated normal control, we have also checked the density of RGCs in sham operated eyes (optic nerve was only exposed without crush). The results showed no differences between normal control and sham operated eyes in the density of RGCs (P = 0.22, 2280 ± 416.1 versus 2475 ± 398.0/mm^2^). The density of RGCs was 847.3 ± 172.7/mm^2^ in the TUDCA treated eyes, 783.9 ± 419.3/mm^2^ in the citicoline treated eyes, 91.5 ± 66.0/mm^2^ in the NT-4 treated eyes, 938.8 ± 477.2/mm^2^ in the Doublet-1-treated eyes, 1024 ± 223.3/mm^2^ in the Doublet-2-treated eyes, and 1215 ± 560.8/mm^2^ in the Triplet treated eyes. The densities of RGCs in the citicoline, TUDCA, Doublet-1, Doublet-2, and Triplet groups were significantly higher than in the PBS group (*P* < 0.001) (Fig. 1Aa–h,B). In the Triplet treated eyes, the density of RGCs was significantly higher than that in single drug treated eyes (P = 0.011; citicoline group, P = 0.05; TUDCA group, P < 0.001; NT-4 group). To examine optimal effective doses of drugs, we have also performed Brn3a immunostaining of whole retina treated with combined 1 mM citicoline, 1 mM TUDCA, and 1 ng/ml NT-4 (low dose Triplet) 2 weeks after ONC. The density of RGCs in low dose Triplet treated eyes was 41.99 ± 14.97/mm^2^, which was significantly lower than that in normal Triplet treated eyes (P < 0.0001) and no significant difference was observed between the PBS group and the low dose Triplet group (P = 0.3297) (Fig. 1Ai,B).

### Detection of apoptotic cells in the ganglion cell layer (GCL)

**Figure 2:**
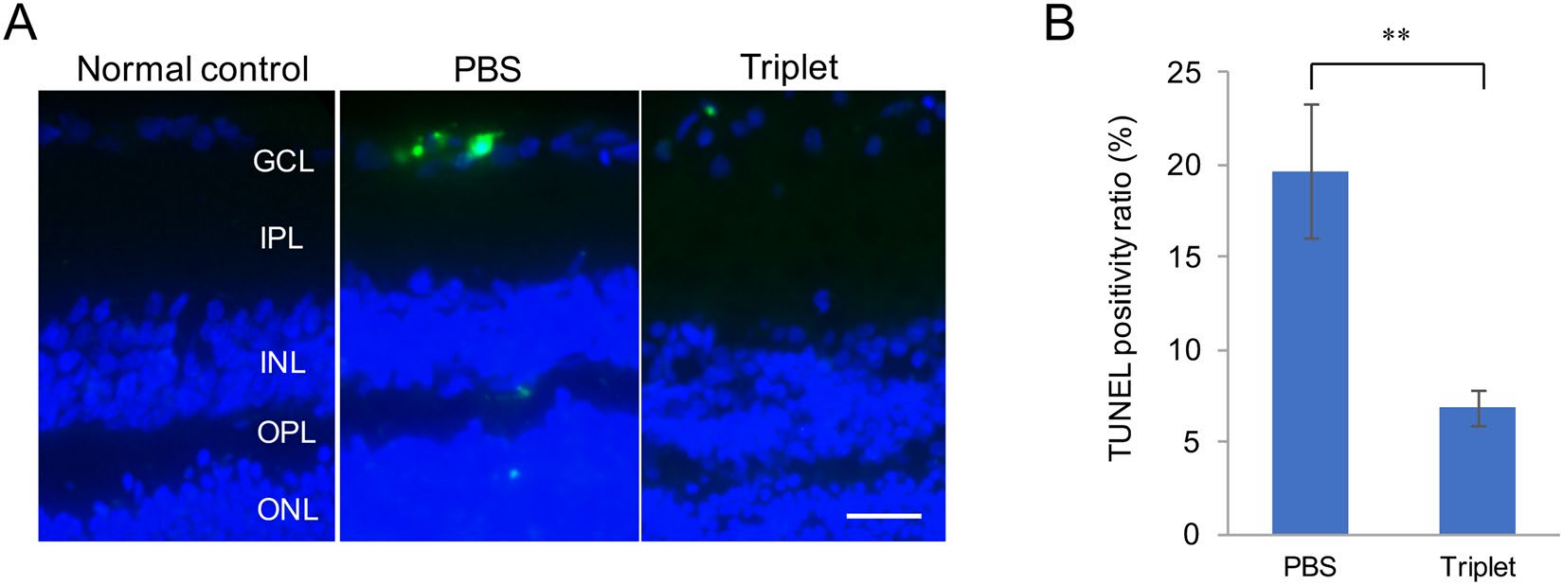
(**A**) Representative photomicrographs of TUNEL positive cells in the ganglion cell layer (GCL) in normal control, PBS and Triplet-treated rats at 5 days after ONC. (Scale Bar: 20 μm) (**B**) Graph showing the ratio of TUNEL-positive to the total number of retinal neuronal cells in the GCL after ONC. Compared with the untreated PBS group, TUNEL positive ratio was decreased significantly in the Triplet eye drop treatment group (**P < 0.001). Values represent means ± SEM of n = 3 animals per experimental group.

To evaluate the status of apoptosis after ONC, we compared the number of apoptotic cells in the GCL between the PBS group and the Triplet treated group by TUNEL staining. In the Triplet treated eyes, the number of TUNEL-positive cells in the GCL was significantly lower than that in the PBS group (6.84 ± 0.97% versus 19.7 ± 3.6%, P < 0.0001) (Fig. 2).

### Neurofilament heavy chain-immunopositive axons in optic nerve

**Figure 3:**
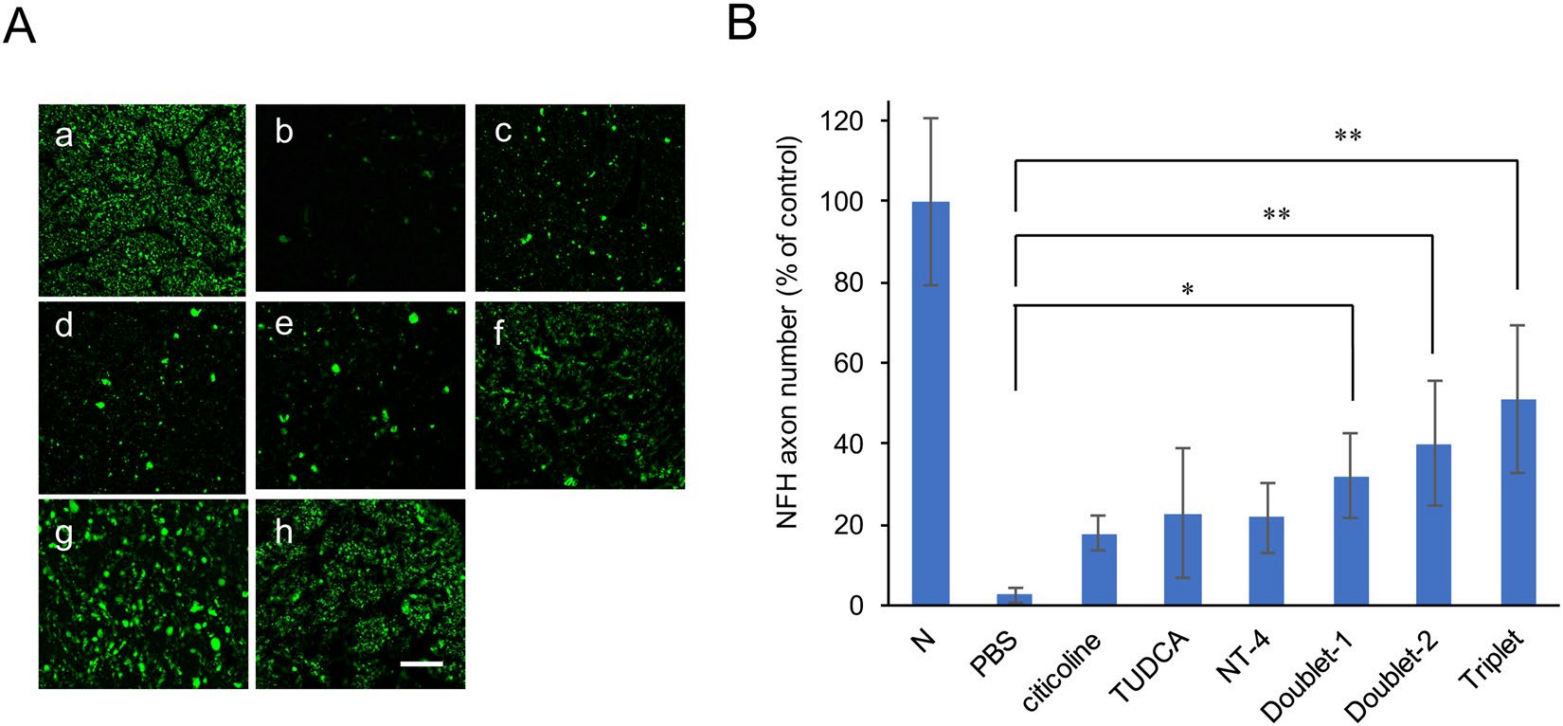
(**A**) Representative photomicrographs of NFH-immunopositive axons in the optic nerve. (a) Normal, (b) PBS, (c) citicoline, (d) TUDCA, (e) NT-4, (f) Doublet-1 (ONC + citicoline + TUDCA), (g) Doublet-2 (ONC + TUDCA + NT-4), (h) Triplet (ONC + citicoline + TUDCA + NT-4). (Scale Bar: 50 μm.) (**B**) Graph showing average number of NFH immunopositive axon counts. Compared with the untreated group, the decrease in axon number was suppressed significantly in the combination eye drop treatment group. (*P < 0.05; Doublet-1 group, **P < 0.001; Doublet-2 group and Triplet group.) Values represent means ± SEM of n = 3–4 animals per experimental group. Significances of intergroup differences were evaluated by one-way ANOVA with Tukey’s multiple comparisons test. N, normal control; Doublet-1, citicoline + TUDCA; Doublet-2, TUDCA + NT-4; Triplet, citicoline + TUDCA + NT-4.

To investigate the neuroprotective effect of topical neurotrophic factors on axonal damage, we counted the number of axons by immunostaining optic nerve sections with anti-200kD neurofilament heavy chain (NFH) antibody two weeks after the ONC. The numbers of NFH-immunopositive axons decreased to 2.6% in the normal control eyes, 8.6% in the citicoline-treated eyes, 17.9% in the TUDCA-treated eyes, 21.7% in the NT-4-treated eyes, 32.2% in the Doublet-1-treated eyes, 40.3% in the Doublet-2-treated eyes, 51.2% in the Triplet-treated eyes (Fig. 3). The number of axons was significantly decreased in each group compared with that of the control (*P* < 0.001). However, the decrease in the axon number was depressed significantly in the combination topical treatment groups (*P* = 0.004; Doublet-1 group, *P* < 0.001; Doublet-2 group, *P* < 0.001; Triplet group). In the Triplet group, the number of NFH-immunopositive axons was significantly higher than all of the single treatment groups (*P* < 0.001).

### Optic nerve regeneration

**Figure 4:**
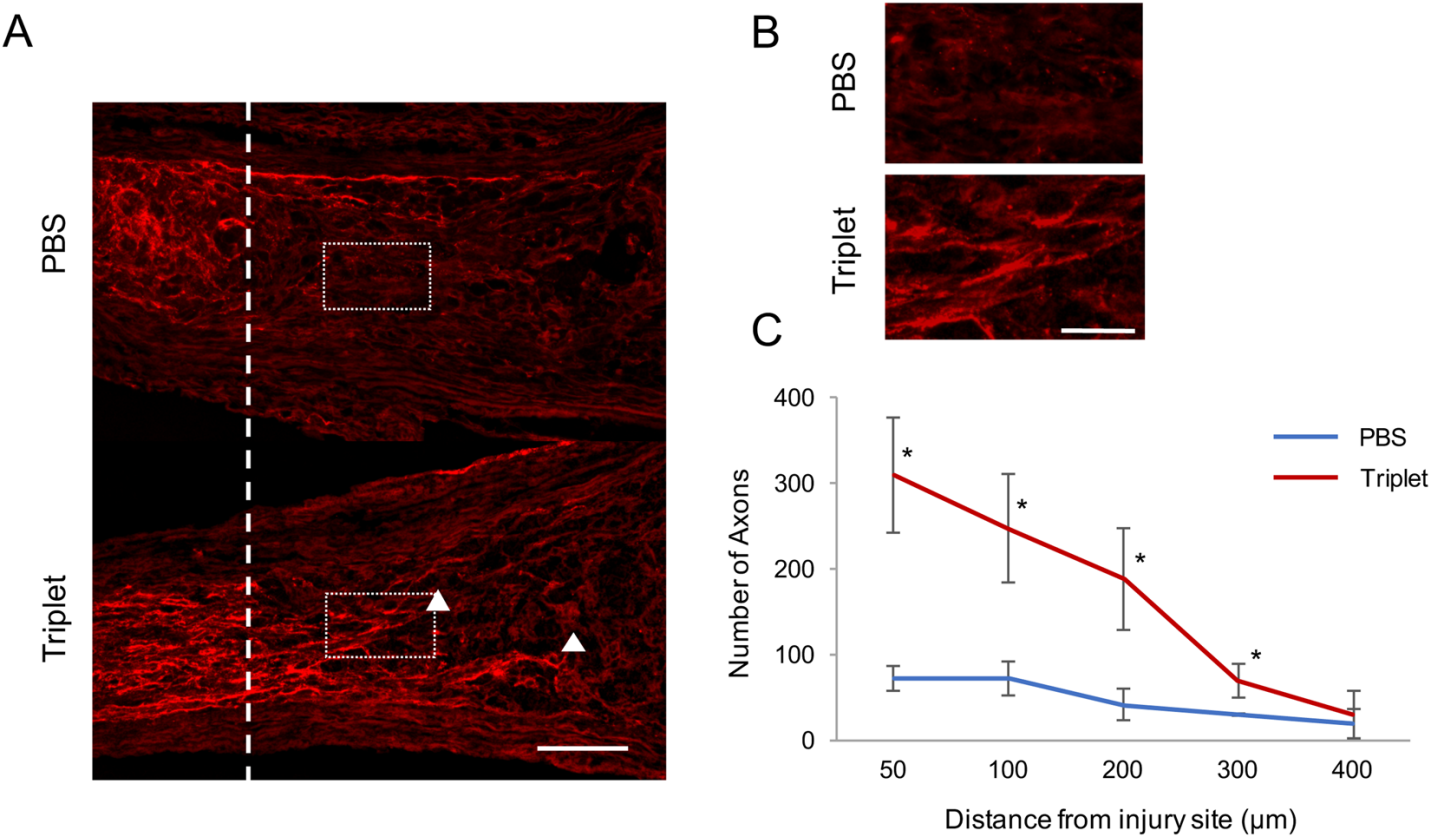
Triplet instillation induces axonal regeneration after ONC. (**A**) Sagittal sections through the optic nerve showing GAP-43-labeled axons distal to the injury site (dotted line) in PBS and triplet-treated rats at 2 weeks after ONC. Arrowheads indicate regenerating axons. (Scale bar:100 µm) (**B**) Higher magnification of the images shown in figure A. (Scale bar:50 µm) (**C**) Graph showing quantification of regenerating axons at different distances distal to the lesion site (*P < 0.05). Values represent means ± SEM of n = 3 animals per experimental group.

Since the Triplet treated eyes have the most protective effect on RGC survival after ONC, we assumed that these combined drugs may possibly have axon regeneration effect. The regenerating axons of RGCs were detected by GAP-43 immunostaining at fixed distances from the injury site. Two weeks after ONC, the number of GAP-43 immunopositive axons in the Triplet treated eyes was greater than that in the PBS groups (Fig. 4A,B). The quantitative analyses showed the number of regenerating axons that extended beyond 200 μm was 42 ± 19.1 in the PBS group compared with 187.3 ± 59.5 in the Triplet group.

### Development of adverse events

**Figure 5:**
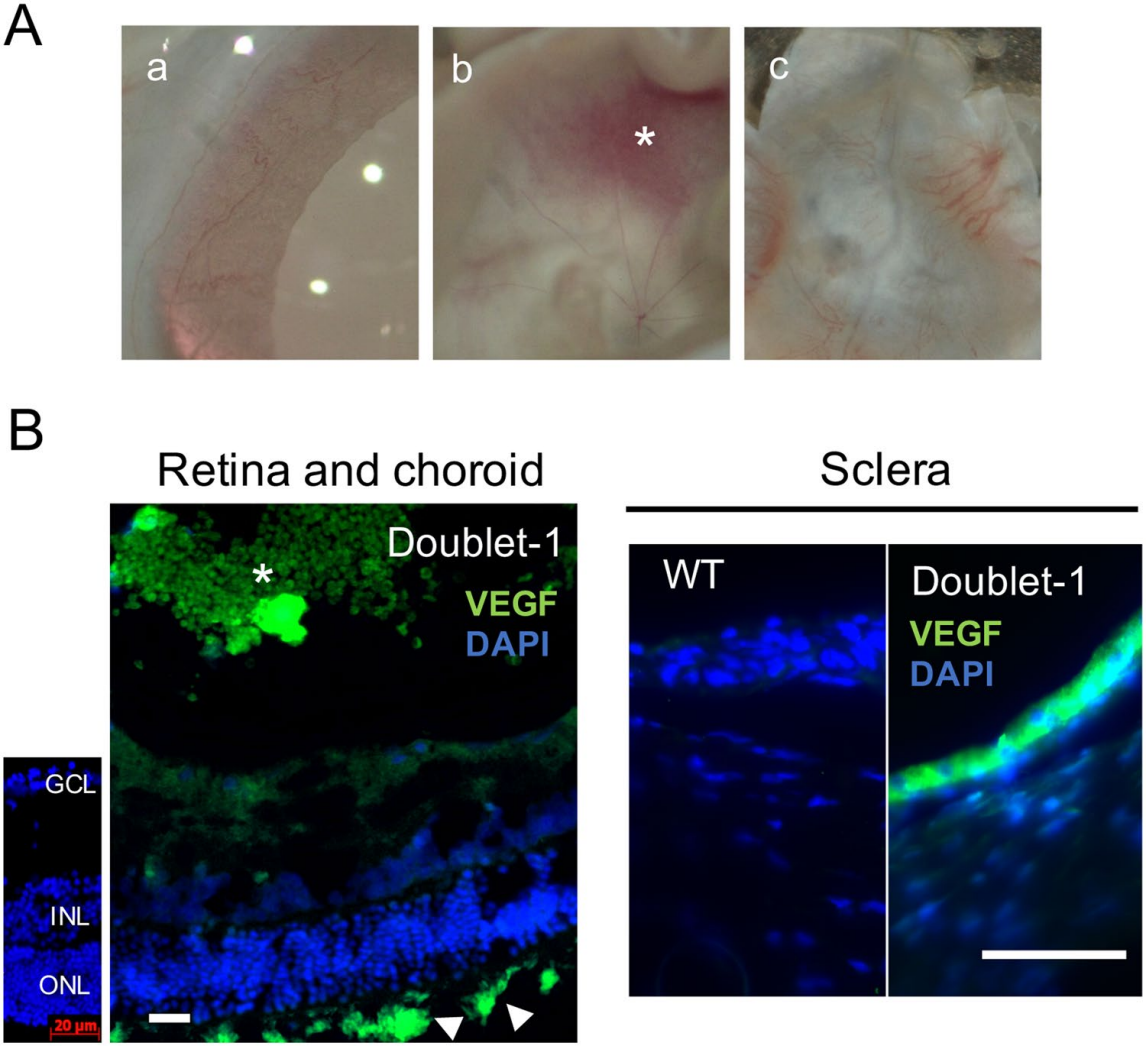
(**A**) Representative photographies of iris, cornea, and sclera in Doublet-1 group. Neovascularizations of the retina, iris, and choroid were observed in 8 of 13 rats (62%) in the Doublet-1 treated eyes. (a) Iris, (b) Retina and epiretinal neovascular membrane (*), (c) Choroid and sclera (**B**) Representative photomicrographs of VEGF-immunopositive sclera and epiretinal membrane in the Doublet-1 treated eye. Intense VEGF immunopositive signaling was observed in the sections of sclera, choroidal vessels (arrowhead) and epiretinal neovascular membranes (*) Scale Bar: 20 μm.

Neovascularization of the cornea, iris, and choroid was observed in 8 of 13 rats (62%) in the Doublet-1 treated eyes **(**Fig. 5A). Intense vascular endothelial growth factor (VEGF) immunopositive staining was observed in the cryosectioned sclera and epiretinal neovascular membranes **(**Fig. 5B**)**. No adverse effects were found in the other groups.

## Discussion

Citicoline is an intermediate endogenous compound, and it promotes the biosynthesis of phospholipids and phosphatidylcholine which are constituents of cell membranes. It has been suggested that citicoline inhibits the degradation of cardiolipin which is a mitochondrial specific phospholipid of inner membranes and is important for mitochondrial function^22,23^. Thus, the neuroprotective effect of citicoline may be as a mitochondrial stabilizer. The results of our earlier animal studies suggested that the anti-apoptotic effect of citicoline was involved in mitochondria-dependent cell death^8^. Citicoline also augments tyrosine hydroxylase activity and increases the dopamine levels by inhibiting dopamine re-uptake. Moreover, citicoline increases the levels of other neurotransmitters, such as acetylcholine, noradrenaline, and serotonin^24,25^. Topical citicoline has already been reported to have neuroprotective effect from the results of human preclinical studies in glaucomatous optic neuropathy^12,13^. Currently, citicoline is marketed as a neuroprotective eye drop in Europe (Citicoline sodium salt: 0.2 g, hyaluronic acid; 0.02 g, Benzalkonium chloride 0.001 g water for injection up to 10 ml, OMK1®, Omikron Italia, Italy, 3 drops/day). Our study supports the existing data showing the neuroprotective effect of citicoline.

TUDCA is a taurine conjugate formed from ursodeoxycholicacid and is known to modulate the function of the endoplasmic reticulum (ER) to protect cells against the apoptosis induced by ER stress^26^. TUDCA reduces ER stress by improving the folding capacity of proteins^27^ and decreasing the level of oxygen free radicals^28^. The results of our earlier study indicated that the neuroprotective and regenerative effects of TUDCA were correlated with the suppression of the expression of p-JNK which is activated under ER stress through IRE1α^5^. *In vivo* studies have shown that TUDCA applied orally or intraperitoneally had neuroprotective effects. In a study of RGC degeneration following an intravitreal injection of NMDA, daily intraperitoneal injections of TUDCA preserved the RGC function as determined by the scotopic threshold response, an ERG component generated by the RGCs^29^. The results of our study support the existing data by showing the neuroprotective effect of TUDCA, and this is the first *in vivo* study showing a neuroprotective effect of TUDCA applied topically.

NT-4 is a member of the nerve growth factor (NGF) family of proteins along with NT-3, brain-derived neurotrophic factor (BDNF) and NGF. NT-4 is believed to act on the tyrosine kinase receptor TrkB and BDNF. Upon binding to NT-4 or BDNF, TrkB forms dimers, activates an intrinsic kinase activity, evokes autophosphorylation, and activates various intracellular signaling cascades including the PI3K/Akt and Ras/ERK1/2 pathways. These pathways play an important role on neuroprotection and axonal regeneration^30–32^. Earlier, we investigated the neuroprotective and regenerative effects of NT-4 on retinal neurons under diabetic conditions. NT-4 increased the rate of surviving cells and regenerating neuronal cells in isolated retinas incubated in high glucose media^4^. The neuroprotective effects of NT-4 were related to the reduction of cascade-9 and -3 activation, the expression of PERK and CHOP, and the expression of c-Jun and JNK^6,7^.

In this study, we showed that crushing the optic nerve led to a loss of RGCs and topical application of three neuroprotective agents in combination for 2 weeks twice a day had neuroprotective and anti-apoptotic effects on RGCs. An adverse effect of simultaneous instillation of 100 mM citicoline and 100 mM TUDCA was the development of neovascularization in the cornea, iris, and choroid and the formation of epiretinal neovascular membranes. There are several studies that have examined the neuroprotective effects of a single neuroprotective agent^10–14^, but this is the first study that examined the neuroprotective effects of a combination these three agents.

In our previous *in vitro* study, NT-4 had the most significant neuroprotective effect on RGCs in AGEs-exposed retinas^9^. However, in this *in vivo* study, no significant neuroprotective effect was found in the NT-4-treated eye. There are two possibilities why the effect of NT-4 instillation was reduced; the drug concentration used in this study (10 ng/ml) was 10 times lower than in the previous *in vitro* study^9^, and the drug concentration at the retina may not have attained a high enough concentration. In fact, after topical application of NT-4, the route from the cornea and conjunctiva to the retina and optic nerve may be reduced because NT-4 is a protein. This differs from that of citicoline and TUDCA which are low molecular weight compounds. However, the results of a recent study showed that topical application of NGF, which is a larger molecular weight protein than NT-4, was effective in protecting RGCs survival and axonal growth two weeks after an ONC in rats^16^. The results of this study indicated that topically applied NGF reached and acted on the structures in the posterior segment of the eye. Thus, it is important to determine the factors that affect the diffusion and transport of neurotrophic factors through the eye structures. In addition, it would be helpful to determine what type of delivery system, e.g., an implantation, microspheres or intravitreous injections would be most effective in delivering NT-4 to the retina.

In the ONC model, the RGC death is preceded by a degeneration of optic nerve due to the crush. Thus, it is important to evaluate the changes in the optic nerve. The results of staining the sections of the crushed optic nerve with NFH, a nerve-specific protein, demonstrated that axonal damage was significantly more suppressed in the combined therapy groups than in the monotherapy group. Furthermore, the Triplet eye drops have an axon regenerating effect probably due to the increased survival of RGCs. Although it may have limited effects on regenerative ability of axons, these results suggest additive effects which enhanced the protection of the axons.

Neovascularization was observed quite regularly only in the combination of citicoline and TUDCA group. It was suggested that VEGF production was induced by the synergistic effect of the combination of citicoline and TUDCA because strong immunopositive signals for VEGF were detected in the sections of the sclera and epiretinal membrane in the Doublet-1 treated eyes. Because neovascularization was not observed in the Triplet group, we suggest that NT-4 may have suppressed the neovascularization. In addition, neovascularization was not noted in the individual drug treated groups. Thus, drugs used in this study seem to be safe for topical administration individually. However, we could not elucidate why only the Doublet-1 treated group developed neovascularization.

The results of a study on an animal model of cerebral infarction showed that citicoline promoted VEGF production in the infarcted area and promoted angiogenesis^33^. Because the optic nerve is a part of the central nervous system, there is a possibility that inflammatory reactions occurred at the time of the ONC, and VEGF production was similarly induced by citicoline instillation. However, neovascularization was not observed with citicoline administration alone, and the relevance of VEGF production by citicoline instillation is still unknown. Contrary to our results, there is a report that VEGF production decreased by one-third after the administration of TUDCA in a laser-induced choroidal neovascularization rat model^34^. Because the ONC is performed by a relatively invasive method and can cause inflammatory reactions by itself, it may be useful to test whether neovascularization develops with citicoline and TUDCA instillation in different optic nerve injury animal models, e.g., NMDA-induced RGCs death model. In addition, it will be necessary to determine whether neovascularization occurs in the untreated group by citicoline and TUDCA instillation. Future molecular-based research should be performed to investigate what type of cells produce VEGF and how the molecular mechanisms of neovascularization develop at high rate in the combination of citicoline and TUDCA group.

There are some limitations in this study. The ONC model does not reflect the characteristics of chronic diseases such as glaucoma, optic nerve diseases, and diabetic neuropathy because it involves acute optic neurodegenerative changes. Our future goal is to determine the neuroprotective effect under the diabetic environment by using a diabetic model. One of challenges of translating the topical application to the clinic is how to make drug delivery of these agents more efficient. Concentrations of therapeutic agent in the anterior chamber and the vitreous body need to be measured to investigate the delivery efficiency of topical application. It has been reported that the intravitreal concentration of citicoline after topical instillation twice a day for three days was an effective concentration^12^. However, there is no report about NT-4 and TUDCA in terms of delivery efficiency after topical application.

In conclusion, the results indicate that neuroprotective effects can be promoted and axonal regeneration can be achieved with topical instillations of combinations of neuroprotective agents. However, we should be aware of possible complications such as neovascularization which occurs in the combination of citicoline and TUDCA. Further studies to identify the cause of adverse effects need to be performed.

## Materials and Methods

### Animals

Seven to 10-week-old male Sprague-Dawley (SD) rats (Japan SLC Co., Hamamatsu, Japan) were used. All of the procedures were performed in accordance with the ARVO Statement for the Use of Animals in Ophthalmic and Vision Research. The experimental protocol was approved by the Ethics of Animal Experiments Committee of Chiba University.

### Optic nerve crush (ONC)

ONC was performed on the left eye of rats under general anesthesia by an intraperitoneal injection of a mixture of ketamine (40 mg/kg/body weight) and xylazine (4 mg/kg/body weight). After anesthesia, an incision was made along the midline of the skull to expose the superior surface of the left eye. The optic nerve was exposed by removing the superior rectus muscle. The optic nerve was crushed with a microvascular clip (Sugita Titanium Aneurysm Clip II) 1 mm posterior to the eye for 10 second. Care was taken not to cause retinal ischemia, and this was confirmed by indirect ophthalmoscopy through dilated pupils. Forty-two rats were divided into 7 groups with 6 animals in each group. The drugs examined were; 100 mM TUDCA, 100 mM citicoline, 10 ng/ml NT-4, combined TUDCA and citicoline (Doublet-1), combined TUDCA and NT4 (Doublet-2) and combined TUDCA, citicoline and NT-4 (Triplet), and PBS. The right eyes were used as control. One drop of each neurotrophic factor was instilled topically immediately after the crushing and then every 12 hours for 14 days. After 2 weeks, the number of surviving RGCs was determined by immunostaining flat mounts of retinas with anti-Brn3a antibody (Santa Cruz Biotechnology, Inc, CA). Cryosections of the optic nerve were immunostained for the anti-200 kD NFH antibody (Abcam, Japan) and anti-GAP-43 antibody (Merck Millipore, USA) to study optic nerve regeneration after optic nerve crush. We also studied apoptotic status in the GCL by TUNEL staining 5 days after ONC. To examine the adverse effects, cryosections of the sclera and the epiretinal neovascular membranes were immunostained for VEGF (1:1000; Santa Cruz Biotechnology, Inc, CA).

### Brn3a-labeled flat mount retina immunohistochemistry (IHC)

Retinal flat mounts were immunofluorescent stained for Brn3a as described in detail^35^. Animals were euthanized by an overdose of isoflurane, and eyes were enucleated, the retinas were isolated, flattened, and fixed in 4% paraformaldehyde (PFA) for 1 hour at room temperature. The flat mounts were then washed with phosphate buffered saline (PBS) 3 times, blocked with blocking buffer (0.1 M PBS with 3% bovine serum and 5% goat serum) for 1 hour, then incubated with mouse anti-Brn3a monoclonal antibody (1:100; Santa Cruz Biotechnology, Santa Cruz, CA, USA) for at least 24 hours at 4 °C. The tissues were then incubated with fluorescein isothiocyanate-conjugated anti-mouse IgG at 4 °C overnight. The number of Brn3a immunopositive cells was counted in four areas randomly selected at a distance of 2 mm from the optic disc (330 μm × 450 μm). The mean number of RGCs/mm^2^ was calculated.

### TUNEL Staining

Five days after ONC, Rats were given an overdose of general anesthesia and were perfused transcardially with PBS followed by 4%PFA. Eyeballs were dissected immediately and immersion-fixed 4%PFA for 2 hours. Retinas were transferred 30% (wt/vo) sucrose for at least 24 hours, embedded in an optimal cutting temperature compound for frozen tissue specimens (Tissue-Tek OCT compound; Sakura Finetechnical Co. Ltd., Tokyo, Japan), and sectioned with a cryostat. TUNEL staining was performed with an apoptosis detection kit (Chemicon International, Temecula, CA) following the manufacturer’s instruction. Hoechst 33342 nucleic acid was used (1:1000) for nuclear counterstain. For quantitative analyses, the ratio of the number of TUNEL-positive cells to the total number of nuclear staining in the GCL was determined.

### Immunohistochemical staining of optic nerves

Fourteen days after ONC, Rats were given an overdose of general anesthesia and were perfused transcardially with 4%PFA. Segments of the optic nerves (8–10 mm) between optic nerve head and optic chiasma were collected. The nerves were post-fixed in 4%PFA at 4 °C for 24 hours, washed with PBS, and immersed in 30% sucrose for at least 24 hours at 4 °C. The optic nerves were embedded in an OCT compound. For GAP-43 immunostaining, optic nerves were Cryostat-sectioned longitudinally at 14 μm, mounted on glass slides. After blocking the sections in 0.1 M PBS with 3% bovine serum and 5% goat serum at RT for 1 hour, they were incubated with the anti-GAP-43 (1:2000; Merck Millipore, USA) at 4 °C overnight, followed by fluorescently labeled secondary antibody. Axons were counted at fixed distances from the injury site in 3 sections per nerve to estimate the total number of regenerating axons^36^. For 200 kD NFH immunostaining, optic nerves were cross-sectioned at 14 μm, mounted on glass slides. To determine the numbers of NFH immunopositive axons in each group, each immunostained sample was used for axon counting. For that purpose, 10–12 sections per group were used. Sections were prepared at the 0.5 mm distal site from the crush level. The fluorescent images were photographed with a fluorescence microscope with a 40X objective lens. The number of axons was determined in at least four areas of 36,960 μm^2^ (165 μm × 224 μm) from each eye. The acquired images were quantified by the Image J software^37^. We have counted the number of the minimal units of axonal bundles in the areas. After being quantified by Image J software, dot-like signals were disappeared. Thus, nonspecific signals such as dot-like staining were not included in this analysis.

### Statistical analyses

All measurements were performed in a masked fashion. Statistical analyses were performed by one-way analysis of variance with Tukey’s multiple comparisons tests for comparisons between each group. Data are presented as the means ± standard deviation (S.D). A *P* < 0.05 was considered statistically significant.
